# Editorial: Discovery of Novel Molecules for Corrosion Protection Using Computational Chemistry

**DOI:** 10.3389/fchem.2018.00277

**Published:** 2018-07-10

**Authors:** Ime B. Obot, Eno E. Ebenso, Duy Q. Dao

**Affiliations:** ^1^Center of Research Excellence in Corrosion, Research Institute, King Fahd University of Petroleum and Minerals, Dhahran, Saudi Arabia; ^2^Department of Chemistry and Material Science Innovation and Modelling Research Focus Area, Faculty of Agriculture, Science and Technology, North-West University, Mmabatho, South Africa; ^3^Institute of Research and Development, Duy Tan University, Da Nang, Vietnam

**Keywords:** corrosion, corrosion inhibitors, computational chemistry, density functional theory, QSAR, molecular dynamics simulations

Several experimental techniques and modern surface characterization tools have been developed to evaluate and characterized the performance of corrosion inhibitors for metals and alloys (Salasi et al., 2007; Lopez and Frankel, 2014; Dwivedi et al., 2017; Obot and Eduok, 2017; Obot et al., 2017). These methodologies are often expensive and tedious, and many are deficient in determining the mechanism of inhibition of the inhibitor molecule with the metal surface at the nanoscale which could be useful in further inhibitor design. The development of density functional theory (DFT) and force field molecular dynamics (MD) simulations approaches provide potential solutions. These approaches involve the use of computational chemistry methods, such as DFT and MD simulations to set up a quantitative structure activity relationship (QSAR), revealing hitherto unrecognized connections between structure and properties which is essential for inhibitor new design and development (Obi-Egbedi et al., 2011; Ghailane et al., 2013; Kabanda et al., 2013; Verma et al., 2018).

The pioneering works of Hohenberg Kohn and Kohn (1964) and Kohn and Sham (1965) have made DFT one of the more popular tools among computational chemical methods. DFT focuses on the electron density, ρ(r), as the carrier of all information in the molecular (or atomic) ground state, rather than on a single electron wave function. Furthermore, studies by Parr, Yang, Chattaraj, Iczkowski, Margrave, Koopmans, Fukui, Pearson, and Sventpaly (Koopmans, 1933; Iczkowski and Margrave, 1961; Pearson, 1963; Fukui, 1982; Yang and Parr, 1985; Parr and Chattaraj, 1991; Parr et al., 1999), have elucidated the so-called “conceptual DFT” in which several molecular parameters and descriptors widely used in molecular characterizations of corrosion inhibitors effectiveness on metal surfaces have been derived.

QSAR approach has been extensively employed in other research fields such as pharmacology (in drug design/development) and toxicology, but its use in the field of corrosion research is not widespread (Zhao et al., 2014). The most important factor that makes the QSAR approach the method of choice for corrosion inhibitor design and development is that it only depends on the molecular structure as opposed to experimental properties of the molecule (Berhanu et al., 2012). Thus, the combination of DFT, force field MD simulations and a QSAR approach could be a very powerful tool in next-generation corrosion inhibitor screening, design, and development.

The above consideration was the motivation for the proposal of this Research topic that aimed at using computational chemistry as a modern tool in the discovery of the “next generation” novel molecules for the protection of metals and alloys of engineering importance. Original research, short communications and review papers all focusing on the use of Computational Chemistry as a modern tool to develop new corrosion inhibitor for corrosion mitigation were expected from contributors. We report in this e-book contribution from 24 authors from the most prestigious research institutions from different parts of the world.

Recently, an excellent review has been published on the use of natural amino acids as corrosion inhibitors (El Ibrahimi et al., in press). However, corrosion inhibition effectiveness of amino acid in acidic medium is pH dependent. Daniel Glossman-Mitnik, Mexico and Juan Frau, Spain conducted a conceptual DFT study for 20 natural amino acids with different ionizable (pH) states using the latest Minnesota family of density functionals (Frau and Glossman-Mitnik). The data obtained from this study indicated that amino acids with a COO– side-chain group (Asp and Glu), together with Lys are the best candidates for the design of small peptides with potential to perform as corrosion inhibitors.

The discovery of volatile corrosion inhibitors (VCI) are often carried out by trial and error and tedious experimental approaches. However, a team led by Assis V. Benedetti from three prestigious Brazillian research institute in Brazil, investigated the possibility of using caprylate salts based on amines as volatile corrosion inhibitors for metallic zinc using both experimental and computational chemistry approaches (Valente et al.). Data obtained from the study indicate a strong correlation between experimental and theoretical results and showed that the molecular size of the caprylate salts is the determining factor in the inhibition efficiency. The models used and experimental results indicated that dicyclohexylamine caprylate is the most efficient inhibitor.

In another contribution, the corrosion study of mild steel in aqueous sulfuric acid solution using 4-Methyl-4H-1,2,4-Triazole-3-Thiol and 2-Mercaptonicotinic Acid were investigated using experimental and Computational Chemistry approaches (Mehmeti and Berisha). The research was conducted in the Department of Chemistry, Faculty of Natural and Mathematic Sciences, University of Prishtina, Pristina, Serbia. The goal of the study was to use theoretical calculations to better understand the inhibition. Experimental and theoretical results showed that 2-mercaptonicotinic acid exhibited the best inhibition efficiency due to the presence of the pyridine nitrogen, carboxyl, and the sulfur atom in the thiol group interacting in a planar geometry with the surface.

Three thiophene derivatives abbreviated as InhA, InhB, and InhC were chosen to theoretically analyze their anticorrosive efficiencies. The work was conducted jointly by a group led by Guo from three different Universities namely in China, Palestine, and in Turkey (Guo et al.). The objective of their work was to assess the anti-corrosive performances of studied molecules by applying quantum chemical calculations (DFT), molecular dynamics simulation, principle component analysis (PCA) as well as agglomerative hierarchical cluster analysis (AHCA). DFT, molecular dynamic simulation, PCA, and AHCA results showed that corrosion inhibition efficiency ranking of studied molecules were as follows InhA > InhB > InhC. The theoretical results were in agreement with experimentally determined inhibition efficiencies of the molecules previously reported.

Finally, a very interesting paper on the combination of virtual screening protocol by in silico toward the discovery of novel 4-Hydroxyphenylpyruvate dioxygenase (HPPD) inhibitors was contributed by a team from China. In the work, an integrated virtual screening protocol combining 3D-pharmacophore model, molecular docking and molecular dynamics (MD) simulation was established to find novel HPPD inhibitors from four commercial databases (Fu et al.). The results provided useful information for developing novel HPPD inhibitors, leading to further understanding of the interaction mechanism of HPPD inhibitors. This present contribution although not focused on corrosion, presented an important contribution of computational chemistry in the area of drug discovery.

## Author contributions

IO wrote the introduction, EE summarizes the papers and DD summarizes and edited the final draft.

### Conflict of interest statement

The authors declare that the research was conducted in the absence of any commercial or financial relationships that could be construed as a potential conflict of interest.
